# Prediction of T-cell epitopes for designing a reverse vaccine against streptococcal bacteria

**DOI:** 10.22099/mbrc.2018.28775.1308

**Published:** 2018-03

**Authors:** Samira Ebrahimi, Hassan Mohabatkar

**Affiliations:** Department of Biotechnology, Faculty of Advanced Sciences and Technologies, University of Isfahan, Isfahan, Iran

**Keywords:** Epitope prediction, Molecular docking, M protein, Streptococcal bacteria, Vaccine design

## Abstract

Streptococcal bacteria are among dangerous human pathogens with major prevalence worldwide. A good vaccine against streptococcal bacteria should have epitopes that confer protection from infection by different streptococcal bacteria types. we aimed was to recognize the most immunogenic and conserved epitopes of streptococcal bacteria, which could be a potential candidate for vaccine development. Nineteen different M proteins of different streptococcal bacteria were chosen and analyzed. Nine-mer epitopes able to simulate both cells mediate and humoral immunity were predicted. Molecular docking was applied in order to measure free binding energy of selected epitopes. Final epitopes were analyzed if they were conserved among different streptococcal bacteria. The identified epitopes require experimental validation for their potential use in peptide vaccines.

## INTRODUCTION

Streptococcal bacteria belong to the phylum Firmicutes and the order lactobacillus [1]. Streptococcal bacteria are among the most important human pathogens responsible for a large variety of diseases among humans ranging from neonates to the elderly [2]. Group A streptococci cause at least 517000 deaths because of severing invasive diseases, 616 million cases of pharyngitis, and 111 million cases of pyoderma [3]. Group B streptococci can cause a wild range of diseases including meningitis, pneumonia, and sepsis. Direct mother-to-baby transmission of the pathogen is the main cause of infections in neonates [4]. Other groups of streptococcal bacteria cause different dangerous human diseases such as brain abscess, pulmonary abscess, and severe infections. 

In establishing infection in the host, an initial and crucial step is the attachment of bacteria to the mucosa and dermal membranes [5]. The main role of bacterial surface proteins is to interact with host cells. Hence, these proteins are potential targets for designing a vaccine against bacterial infections and diseases [6]. M protein is one of the major virulence factors of invasive streptococcal bacteria isolates [7]. This protein is a surface protein that contains two polypeptide chains. M protein has a sorting part in carboxy-terminal that is composed of LPXTG motif required for the exact attachment of M protein onto the cell wall [8]. M proteins have an affinity for albumin, plasminogen, fibrinogen, IgG, IgA, and C4b-binding protein [9] and they have a crucial role in the variety of diseases that can be evoked by streptococcal bacteria [10]. Following the work of Rebecca Lancefield that proved antibodies against the N-terminal of M protein could bring protection in a specific manner, vaccine development against streptococcal bacteria focused on M protein [11].

To reduce costs and experimental efforts in vaccine design, prediction of potentially immunogenic epitopes in a given protein would be a useful way [12]. In the development of vaccines against pathogens, the epitope-driven vaccine is an attractive concept [13]. In these vaccines, selected epitopes should be conserved across pathogen variants and their stages. Also, selected epitopes should evoke the immune response in the desired way. Epitopes ideally must cover a major population and have a binding affinity with more than one major histocompatibility complex (MHC) allele [14].

In this study, a reverse vaccinology approach was applied for epitope prediction of different M proteins of streptococcal bacteria. Epitope prediction is based on predicting epitopes that stimulate both humoral and cell-mediated immunity. Molecular docking was applied to model the interaction between the final epitopes with HLA-A1 allele. Conservation of peptides among studied M proteins was checked. Finally, selected epitopes were checked if they were promiscuous epitopes, which means they could be epitopes for several MHC class I and II alleles.

## MATERIALS AND METHODS

**Selection of sequences: **The sequence of 19 different M proteins from different streptococcal bacteria, from NCBI and UniProt were selected. M proteins with their accession numbers are shown in Table 1.

**Table 1:** Selected M proteins of streptococcal bacteria with their accession number

**Name of Bacteria**	**M protein**	**Accession Number**
*agalactiae Streptococcus*	M protein repeat protein	KLL29245.1
*S. pyogene*	Antiphagocytic M protein, type 3	WP_011055052.1
*S. pyogenes*	Antiphagocytic M protein Emm	WP_014407886.1
*S. pyogenes GAS*	M protein type 1	NP_269973.1
*S. pyogenes*	M protein, serotype 5	WP_023079553.1
*S. pyogenes*	M12_STRPY M protein	P19401
*S. pyogenes*	M protein, serotype 24	WP_032465389.1
*S. equi subsp.* *zooepidemicus*	M-protein	AGV40770.1
*S. equi*	M-protein	AAB71984.1
*S. equi subsp.*	M-protein	KED03515.1
*ruminatorum*		
*S. sp. 'group G'*	M protein	AAA26928.1
*S. canis*	M protein	WP_003045324.1
s.* dysgalactiae*	Antiphagocytic M protein	WP_015016601.1
*S. pyogenes*	M protein, serotype 6	WP_023610762.1
*S. pyogenes*	M protein, serotype 49	WP_023612186.1
*S. parauberis*	M protein	WP_004347161.1
*S. pneumoniae*	M protein repeat family protein	WP_001854666.1
*S. pyogenes*	M protein	WP_011529059.1
*S. dysgalactiae*	M protein, serotype 24	WP_048327566.1

**T-cell epitope prediction:** In the present investigation, MAPPP [15], ComPred [16], HLArestrictor [17], MHCPred [18], NetMHC [19], ProPred I [20], RANKPEP [21], SVMHC [22], Vaxitope [23], and IEDB [24] were used as epitope prediction tools. The HLA-A1 restricted 9-mer epitopes of each M protein were predicted by the above-mentioned tools. For each tool and each M protein, top 10 predicted epitopes were selected, and those recognized by at least five tools were selected for further analysis.

**Applying filters B-Cell epitope prediction: **The main purpose of this part of the investigation was to select 9-mer T-cell epitopes, which were part of 20-mer linear B-cell epitopes. Selected epitopes can stimulate both humoral and cell-mediate immunity. ABCpred software (www.imtech.res.in/raghava/abcpred) was used in order to predict antigenicity linear non-overlapping 20-mer B-cell epitopes [25]. B-cell epitopes with score> 0.6 were analyzed.

**Antigenicity of the epitopes: **VaxiJen (www.ddgpharmfac.net/vaxijen) was used to identify if epitopes are antigenic. vaxiJen classified antigens based on the physicochemical properties of proteins [26]. Epitopes with VaxiJen score >0.7 were selected.

**Binding affinity analysis: **Binding affinity was predicted by means of MHCPred server. The results of binding affinity prediction between HLA-A1 allele and selected epitopes were given in terms of inhibitory concentration [IC50] [18]. Epitopes with the IC50 value less than 1000 nm for HLA-A1 were selected.

**Molecular docking of the epitopes binding to HLA-A1: **The 3D structure of selected epitopes binding to HLA-A1 was needed in order to do molecular docking. The 3D structure of selected epitopes was designed using PEPstr server, which predicts the tertiary structure of peptide from the sequence given as input [27]. In addition, the 3D structure of HLA-A1 was retrieved from protein data bank (PDB ID: 4NQV). Epitopes were docked into HLA-A1 allele using ClusPro. The ClusPro program is one of the widely used tools for protein-protein docking [28].

**Selected T-cell epitopes conservation: **Final epitopes were checked if they were conserved among different studied M proteins.

**Selection of promiscuous epitopes: **In the last step, conserved epitopes were checked if they were promiscuous. Promiscuous T-cell epitopes are those, which could bind to different MHC class I and II alleles. ProPred I was used as an MHC class I epitope prediction server [20], and ProPred was used to predict MHC class II epitopes [29].

## RESULTS

Top 10 T-cell epitopes of each of 10 servers recognized by at least 10 servers were identified. The numbers of selected epitopes for each protein are shown in Table 2. In order to select final practical epitopes, different filters were applied. B-cell epitopes with score >0.6 were predicted in order to find epitopes, which stimulate both humoral and cell-mediated immune responses. Antigenicity of the epitopes was measured by VaxiJen server, and epitopes with score <0.7 were omitted. Binding affinity between epitopes and HLA-A1 allele was measured by MHCPred server, and epitopes with IC50 >1000 nm were selected for each protein. Molecular docking between epitopes and HLA-A1 alleles was applied and they showed negative free binding energy in all cases. Different features of final epitopes, which fulfill all mentioned criteria, are shown in Table 3. 

Conservation of the selected T-cell epitopes of each M protein among other M proteins was checked. 9 epitopes were conserved among studied M proteins, as listed in Table 3. The final step of this investigation is to find out if final conserved epitopes are promiscuous. The numbers of MHC class I and II alleles bound to final epitopes are shown in Table 4.

**Table 2:** Number of predicted epitopes by at least 5 servers for each M protein

***Name of bacteria***	**M protein**	**Number of Predicted T-cell Epitopes**
*S. agalactiae*	M protein repeat protein	28
*S. pyogenes*	Antiphagocytic M protein, type 3	39
*S. pyogenes*	Antiphagocytic M protein Emm	30
*S. pyogenes M1 GAS*	M protein type 1	27
*S. pyogenes*	M protein, serotype 5	22
*S. pyogenes*	M12_STRPY M protein	31
*S. pyogenes*	M protein, serotype 24	29
*S. equi subsp. zooepidemicus*	M-protein	25
*S. equi*	M-protein	34
*S. equi subsp. ruminatorum CECT 5772*	M-protein	38
*Streptococcus sp. 'group G'*	M protein	34
*S. canis*	M protein	27
*S. dysgalactiae*	Antiphagocytic M protein	38
*S. pyogenes*	M protein, serotype 6	36
*S. pyogenes*	M protein, serotype 49	34
*S. parauberis*	M protein	31
*S. pneumoniae*	M protein repeat family protein	25
*S. pyogenes*	M protein	35
*S. dysgalactiae*	M protein, serotype 24	30

**Table 3:** Different feathers of final conserved epitopes

**Conserved T- cell Epitope**	**Epitope VaxiJen score**	**No. of methods**	**HLA-A1 (IC50)**	**Binding free energy (Kcal/mol)**	**No. of alleles bounded MHC I+MHC II**
LTEKQNRVS	1.3667	5	371.54	-777.6	1+1
EAELNNLKA	0.8786	7	119.12	-572.3	3+0
IADLETKLK	1.2518	8	115.61	-696.7	1+2
LSDKRHQQE	1.8616	7	56.36	-850.5	1+0
ALEEANSKL	1.0475	9	75.34	-603.2	11+2
ELEAKHQKL	1.4469	7	45.71	-540.9	8+1
ALTAEHQKL	0.8842	5	99.77	-671.1	10+0
LTAEHQKLK	1.8546	6	240.44	-691.4	4+1
GHQHAHNEY	0.9194	5	37.84	-1013.4	3+3

## DISCUSSION

The introduction of genomic technologies such as recombinant DNA technology, in silico analysis, and proteomics revoluted the approach of studying bacterial pathogens and vaccine design. Genomic technologies say that every antigenic part of a pathogen can induce a protective immune response and should be tested to be used in vaccine design [30]. One such approach is reverse vaccinology, in which the mining of genomic and proteomic information of the organism using bioinformatics-based software is done [31].

Initial and crucial step in colonization and establishing infection in the host is the adhesion of bacteria to the mucous and dermal membrane [32]. Surface proteins of pathogens are mostly pathogenic and cause bacterial pathogenicity. Surface proteins are potential targets for vaccine development mostly at preventing bacterial disease and infections [33]. One of the major virulence factors of streptococcal bacteria is M protein, the main role of which is the presentation of opsonophagocytosis [34]. This protein is the favored target of vaccine design [35].

**Table 4:** Conserved epitopes of different studied M proteins

**Conserved Epitope**	**Proteins with conserved epitopes**
LTEKQNRVS	M protein, type 3 of S.pyogenes
	M protein, serotype 24 of *S. pyogenes*
EAELNNLKA	M protein, type 3 of *S. pyogenes*
	M protein, serotype 24 of *S. pyogenes*
	M protein, serotype 12 of *S. pyogenes*
IADLETKLK	M protein, type 3 of *S. pyogenes*
	M protein, serotype 24 of *S. pyogenes*
LSDKRHQQE	M protein, type 3 of *S. pyogenes*
	M protein, serotype 24 of *S. pyogenes*
ALEEANSKL	M protein of *S. dysgalactiae*
	M protein, type 1 of *S. pyogenes*
	M protein, type 5 of *S. pyogenes*
	M protein, serotype 12 of *S. pyogenes*
	M protein, serotype 24 of *S. dysgalactiae*
	M protein of group G
ELEAKHQKL	M protein Emm of *S. pyogenes*
	M protein, type 49 of *S. pyogenes*
ALTAEHQKL	M protein Emm of *S. pyogenes*
	M protein of *S. pyogenes*
LTAEHQKLK	M protein Emm of *S. pyogenes*
	M protein of *S. pyogenes*
GHQHAHNEY	M protein, serotype 12 of *S. pyogenes*
	M protein, serotype 24 of *S. dysgalactiae*

Early studies in vaccine design against streptococcal bacteria were focused on using whole protein [36]. Later studies showed contribution of epitopes to the pathogenicity of bacteria. Consequently, the use of full-length M protein as streptococcal bacterial vaccine candidates was prohibited. Later studies targeted immunogenic amino-terminal region of M proteins [37] and conserved C-terminal region of M protein [38]. Until now, efforts to develop a useful universal vaccine against streptococcal bacteria have not been unsuccessful. Therefore, in this investigation, the main aim was the detection of antigenic conserved epitopes in different M proteins. In order to recognize the most appropriate candidates for vaccine design, different filters were sequentially applied. Finally, selected T-cell epitopes, which are antigenic and capable of inducing antibody production, are detected. The identified epitopes require experimental validation for their potential use in peptide vaccines.

In conclusion, in this work, antigenicity testing, B-cell and T-cell epitope prediction, and molecular docking has been done for different streptococcal M proteins. Our results show that nine different are appropriate candidates for peptide vaccine. Molecular docking results show that these nine peptides have low free binding energy in binding to MHC alleles, which means these peptides are good binders and can simulate the immune response. *In vitro* and *in vivo* immunological tests should be performed in order to validate the suitability of the epitopes for vaccine development.
